# Hiding in plain sight: case report of congenital simple hamartoma of the retinal pigment epithelium

**DOI:** 10.22336/rjo.2026.58

**Published:** 2026

**Authors:** Ivana Ljubić Zeljko, Irena Sesar, Ivan Zeljko, Renato Pejić

**Affiliations:** 1 Department of Ophthalmology, University Clinical Hospital Mostar, Mostar, Bosnia and Herzegovina; 2 Department of Ophthalmology, University of Mostar School of Medicine, Mostar, Bosnia and Herzegovina; 3 Department of Pathophysiology, University of Mostar School of Medicine, Mostar, Bosnia and Herzegovina

**Keywords:** congenital simple hamartoma of the retinal pigment epithelium, pigmented retinal lesions, optical coherence tomography, CSHRPE, CSHRPE = Congenital simple hamartoma of the retinal pigment epithelium, BCVA = Best corrected visual acuity, OCT = Optical coherence tomography, FAF = Fundus autofluorescence, FA = Fluorescein angiography, RPE = Retinal pigment epithelium

## Abstract

Congenital simple hamartoma of the retinal pigment epithelium (CSHRPE) is an extremely rare benign intraocular tumor, originating from the retinal pigment epithelium. The exact prevalence of CSHRPE is unknown, but very few cases have been reported in the literature to date. It is typically asymptomatic, discovered mostly incidentally during routine eye examination, and it can mimic other pigmented retinal lesions, posing diagnostic challenges. We present a case of a 53-year-old woman referred for ophthalmological evaluation due to recurrent, non-specific headaches. Fundus examination revealed a juxtafoveal, hyperpigmented lesion of the left eye with associated drusen around the lesion. Multimodal imaging, including fundus autofluorescence (FAF), optical coherence tomography (OCT), and fluorescein angiography (FA), confirmed the diagnosis of CSHRPE. OCT showed a well-demarcated, elevated, hyperreflective lesion causing optical shadowing of deeper retinal layers. FAF demonstrated a complete lack of autofluorescence at the lesion site, while FA revealed hypofluorescence throughout all phases without autonomous circulation. Unlike prior reports describing complications such as macular edema, epiretinal membrane, or macular hole, no such changes were observed in this patient. The lesion was stable over five years of follow-up, and visual acuity remained unaffected. In the absence of complications or progression, no treatment was necessary, but periodic monitoring is recommended.

## Introduction

Congenital simple hamartoma of the retinal pigment epithelium (CSHRPE) is an extremely rare benign intraocular tumor, originating from the retinal pigment epithelium. These lesions are frequently detected incidentally during routine eye examination because patients are usually asymptomatic. Generally, the lesions do not show signs of progression [1]. However, in some cases, they may present diagnostic challenges due to their similarity to other pigmented retinal lesions, including malignant or metastatic conditions, congenital or acquired hypertrophy of retinal pigment epithelium, etc. [2]. CSHRPE typically presents as solitary or multiple nodular hyperpigmented lesions [3].

The significance of this report lies in the rarity of CSHRPE and the importance of distinguishing it from other conditions that mimic its presentation. Accurate diagnosis is crucial to prevent unnecessary interventions and ensure proper patient management.

## Case presentation

Here, we present a case of a 53-year-old woman who presented for an ophthalmological examination upon referral from her neurologist. Her primary complaint was recurrent, non-specific headache that had persisted for several years. She had no significant ophthalmic or systemic medical history. On examination, her best corrected visual acuity (BCVA) was 1.0. Intraocular pressure was within normal limits, with normal findings of the anterior eye segment bilaterally. The posterior segment of the right eye was unremarkable. Fundus examination of the left eye revealed round hyperpigmentation in the macula with well-defined margins surrounded by several small drusen (**Fig. 1**). Optical coherence tomography (OCT) imaging of the left eye showed a lesion 767 microns in diameter, 496 microns in maximum height, discretely elevated and clearly demarcated from the surrounding retina, showing signs of hyperreflectivity in the inner retinal layers with significant optical obscuration of all distal external structures in the projection of the lesion. Surrounding retina was unaffected (**Fig. 2**). Fundus autofluorescence (FAF) showed a complete lack of autofluorescence (**Fig. 3**). Fluorescein angiography (FA) demonstrated a complete blockage of the background fluorescence in the pre-arterial and early arterial phase in the projection of a clearly demarcated lesion. In the arterial and arterial-venous phase, the blood vessels were properly filled with contrast, while the pigmented lesion showed no signs of autonomous circulation or contrast staining. In the late arteriovenous phase, there was contrast staining of small point lesions next to the macular branches of the upper branch of the central retinal artery, which intensified in the recirculation phase, but without signs of confluence. In the post-contrast phase, late contrast retention occurred in the projection of the described hard drusen. At the same time, the pigmented juxtafoveolar lesion showed signs of hypofluorescence throughout all phases of angiography (**Fig. 4A, B**). B-scan ultrasound did not detect the lesion. Based on the clinical appearance, location of the lesion, imaging findings, and lack of systemic symptoms, the patient was diagnosed with congenital simple hamartoma of the retinal pigment epithelium. Given the benign nature of the lesion and the absence of associated complications, no treatment was required. The patient was reassured and scheduled for routine follow-up to monitor the lesion for any potential changes.

**Fig. 1:**
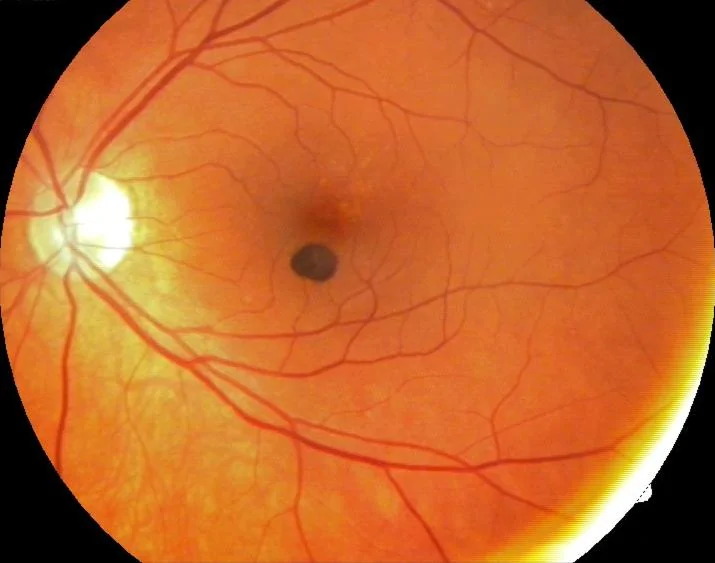
Fundus photograph of the left eye shows a congenital simple hamartoma of the retinal pigment epithelium located juxtafoveal

**Fig. 2:**
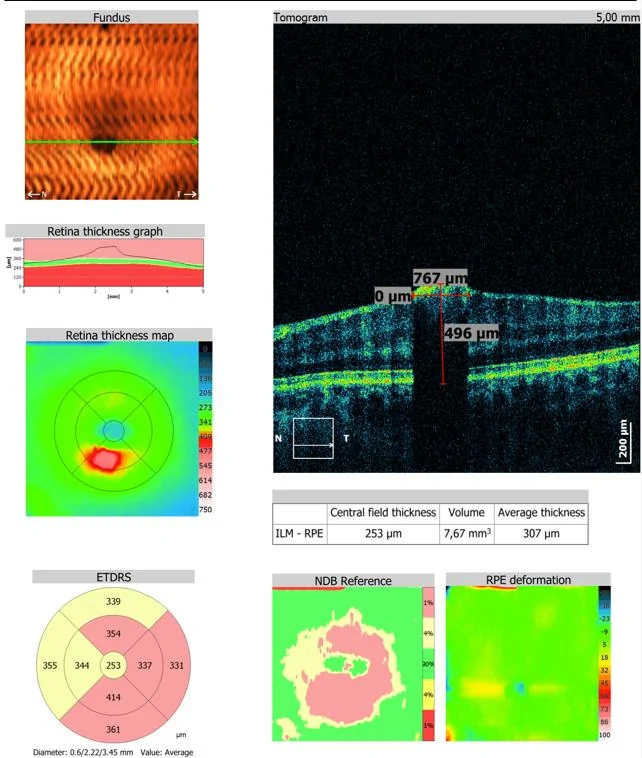
Optical coherence tomography (OCT) scan of the macula of the left eye shows an elevated hyperreflective lesion with a shadow effect

**Fig. 3:**
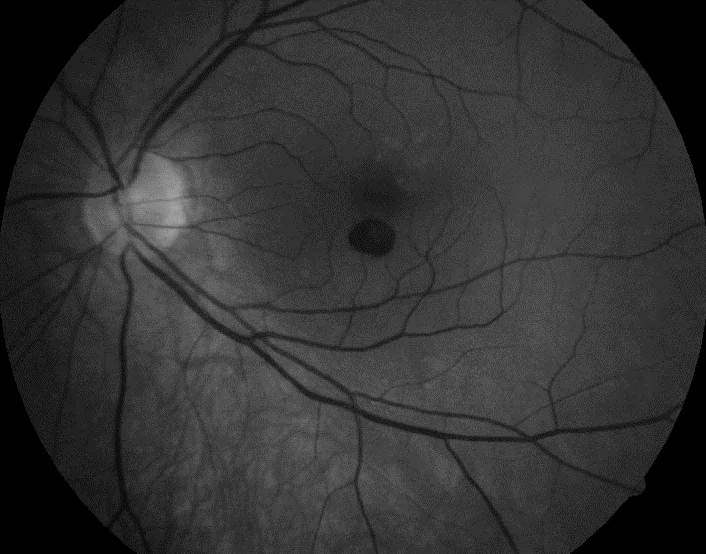
Fundus autofluorescence (FAF) of the left eye shows a complete lack of autofluorescence

**Fig. 4:**
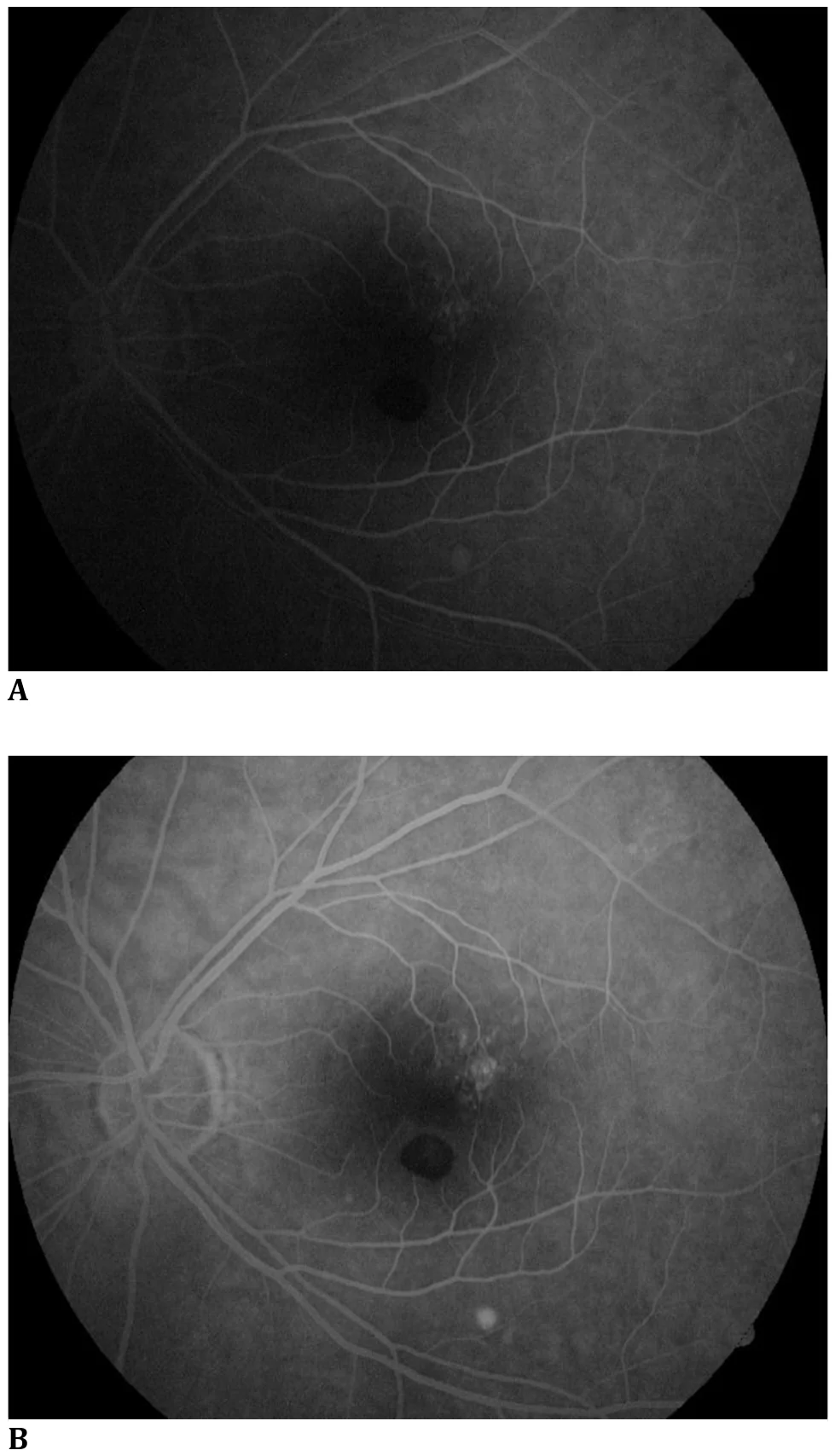
Fluorescein angiography (FA) shows hypofluorescence throughout all phases of angiography in the lesion area (**A, B**) with hyperfluorescence around the lesion in the late phase (**B**)

## Discussion

CSHRPE is a rare, benign intraocular lesion that arises from abnormal development of the retinal pigment epithelium (RPE). These lesions are typically asymptomatic and discovered incidentally during routine fundus examinations [1]. In most cases, this lesion is detected in children and young adults, as is the case in the papers published by Badawi AH and Khadamy J [4,5]. Unlike them, the patient in our case was middle-aged.

In the literature, CSHRPE is often described as a round, black nodular lesion with well-demarcated margins that appears to involve the full thickness of the retina and RPE. Visual acuity is usually normal, as it was in our case, but it can rarely be impaired due to foveal involvement [6,7]. The patient presented in this case had a juxtafoveal location of CSHRPE, which did not cause any complications during 5 years of follow-up. Complications described in previous reports include epiretinal membrane, macular edema, and macular hole formation [6-9].

Due to its rarity and similar clinical appearance, the primary challenge in diagnosing CSHRPE lies in differentiating it from other pigmented lesions, such as congenital or acquired hypertrophy of retinal pigment epithelium, combined hamartoma of the retina and RPE, choroidal melanoma, choroidal nevus, or metastatic carcinoma [2-3]. This case highlights the importance of multimodal imaging in establishing an accurate diagnosis and avoiding overtreatment.

As the main diagnostic test for confirming CSHRPE in our patient, OCT showed an elevated hyperreflective lesion at the surface with optical shadowing of the outer retinal layers, which was identical to the findings of previous reports [1-9]. Fluorescein angiography in our patient revealed hypofluorescence throughout all phases of angiography in the lesion area, along with hyperfluorescence of small hard drusen around the lesion in the late phase, which was similar to what was reported by Shields JA, Shields CL, and Nakatsuka AS et al. [3-4]. In our case, the ultrasound of the eye was unable to detect the lesion, which contrasted with findings reported in other case studies [3]. Fundus autofluorescence of this lesion was first described by Teke MY et al. in 2012. They detected a blockage of background autofluorescence at the site of a heavily pigmented lesion, which was also found in the presented case [10].

One distinguishing feature of this case is the incidental discovery of the lesion during evaluation for headache. While unrelated to the ocular findings, the presenting symptom underscores the importance of thorough ophthalmic evaluation in patients referred for systemic complaints.

## Conclusion

In conclusion, during 5 years of follow-up, our patient’s lesion did not change. It did not cause any complications, so the treatment was not generally necessary, but periodic monitoring is recommended.
